# Supplementary figures and images for: Real-time loop-mediated isothermal amplification for rapid detection of Enterocytozoon hepatopenaei
Source: PeerJ. 2018 Dec 4;6:e5993. doi: 10.7717/peerj.5993 (PMC6284447; doi:10.7717/peerj.5993)

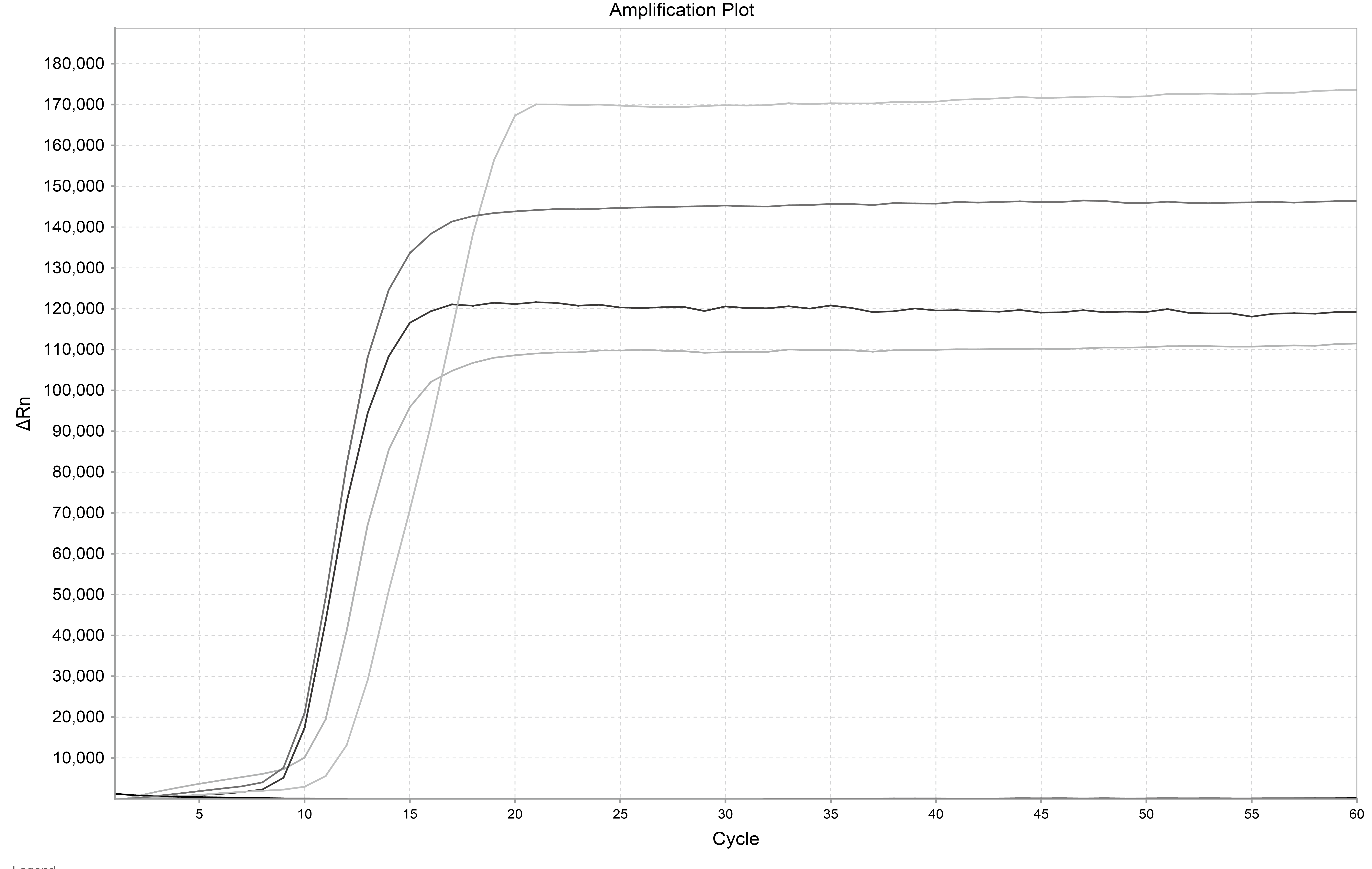

Supplement: Supplemental Information 1 — The ΔRn vs. cycle graph was plotted automatically by StepOne Real-Time PCR System. ΔRn is the fluorescence unit minus the baseline, the graph depicts the ΔRn on the Y axis and cycle number on the X axis. (A) Amplification curve of Enterocytozoon hepatopenaei (EHP) 1. (B) Amplification curve of EHP 2. (C) Amplification curve of EHP 3. (D) Amplification curve of EHP 4. (E) Amplification curve of the negative control. [file peerj-06-5993-s001.png]

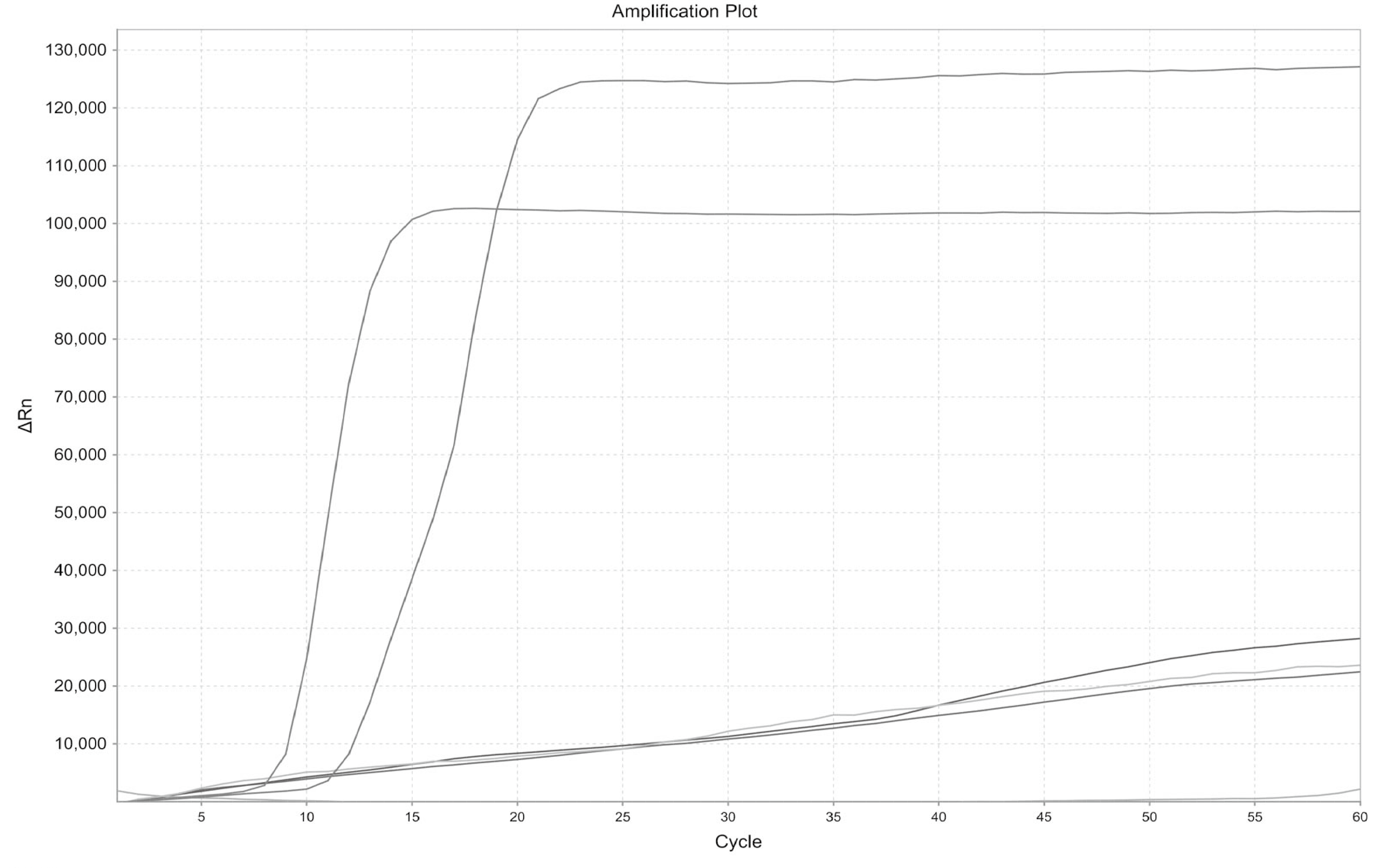

Supplement: Supplemental Information 2 — Three different pathogen templates were performed along with Enterocytozoon hepatopenaei (EHP). (A) Amplification curve of EHP 3. (B) Amplification curve of EHP 4. (C) Amplification curve of white spot syndrome virus (WSSV). (D) Amplification curve of macrobrachium rosenbergii noda virus (MrNV). (E) Amplification curve of infectious hypodermol and hematopoietic necrosis virus (IHHN). (F) Amplification curve of the negative control. [file peerj-06-5993-s002.png]

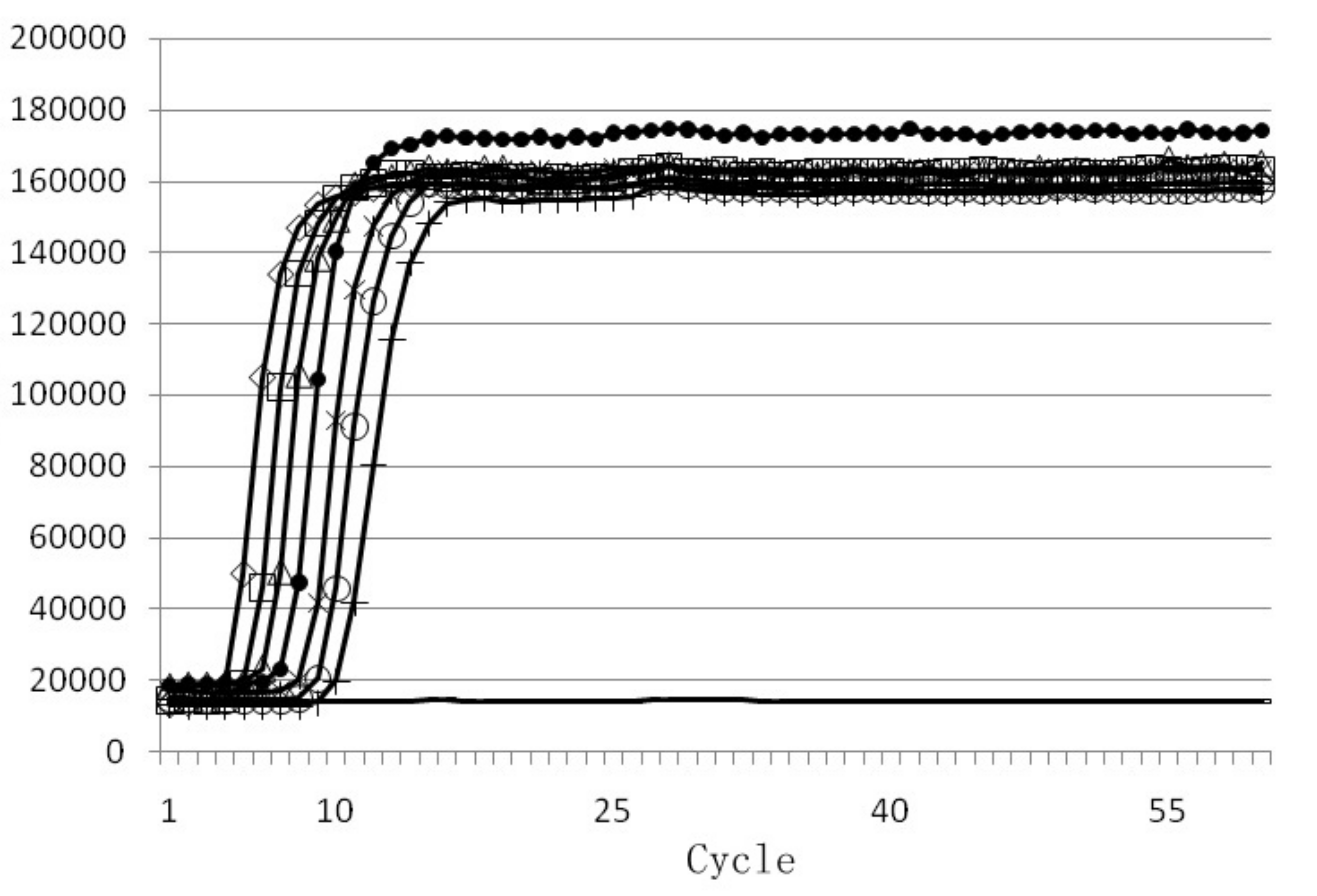

Supplement: Supplemental Information 3 — The fluorescence unit vs. cycle graph was plotted from the data of the StepOne Real-Time PCR System. Serial 10-fold dilutions ranging from 100 times to 10−6 times for Enterocytozoon hepatopenaei (EHP) positive sample DNA. (NG) Negative control. [file peerj-06-5993-s003.png]

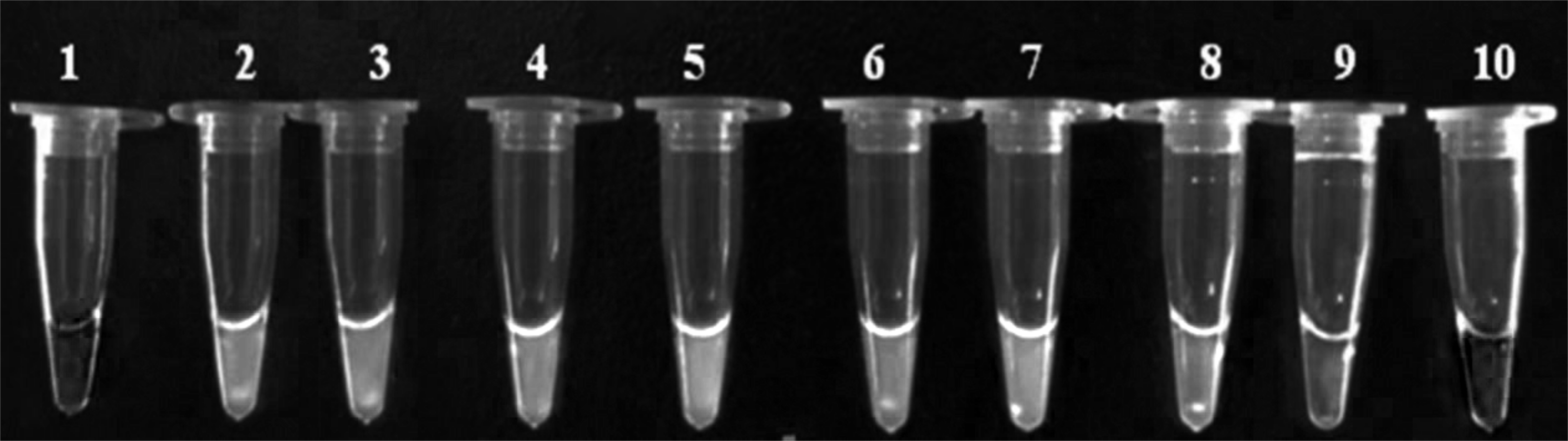

Supplement: Supplemental Information 4 — (A) Negative control. (B) Positive control. (C) Enterocytozoon hepatopenaei (EHP). (D-I) Corresponding to serial 10-fold dilutions ranging from 100 times to 10−6 times for EHP positive sample DNA, respectively. (J) Negative control. [file peerj-06-5993-s004.png]

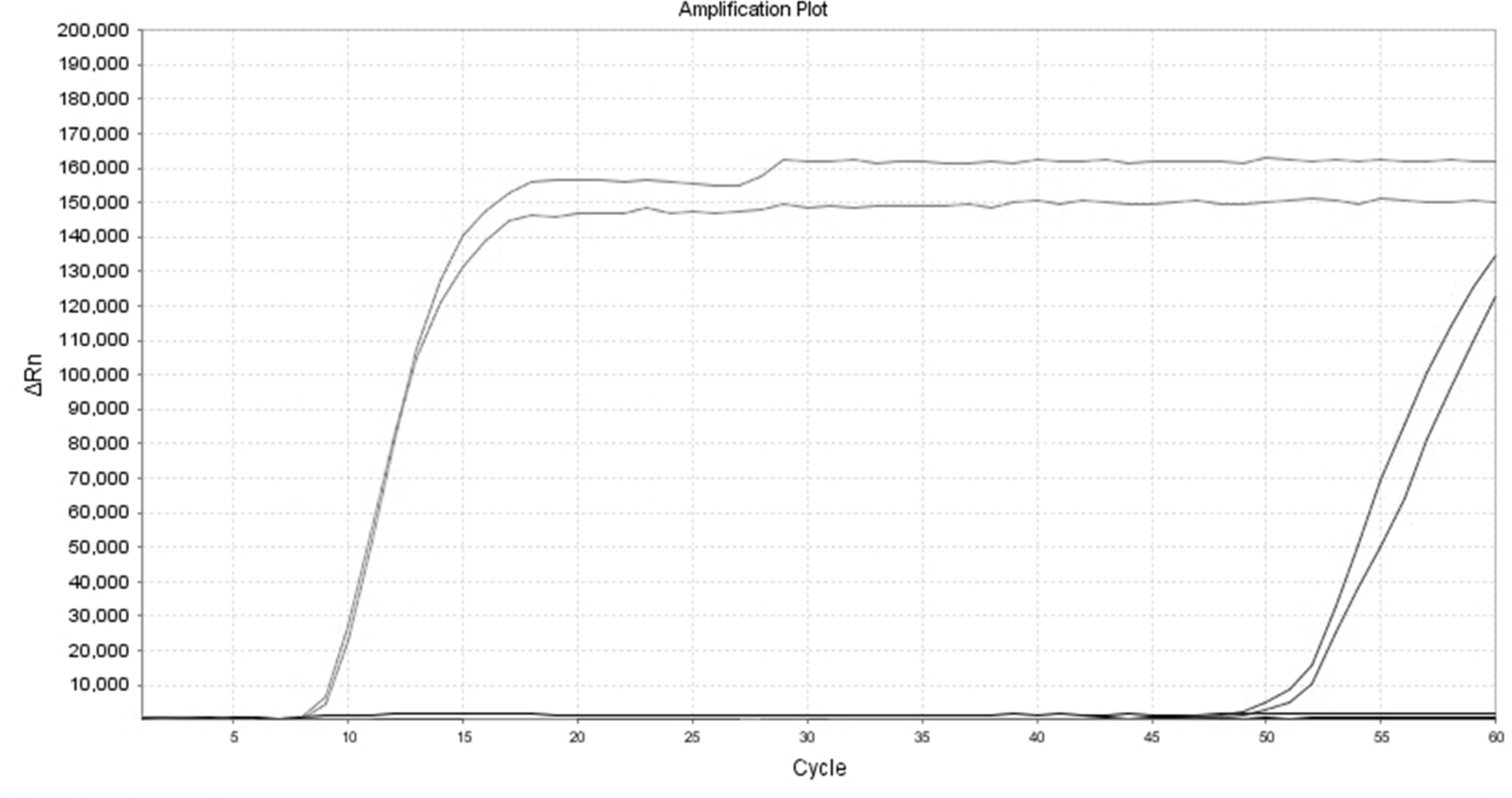

Supplement: Supplemental Information 5 — (A) Amplification curve of real-time Loop-Mediated Isothermal Amplification. (B) Amplification curve of conventional Loop-Mediated Isothermal Amplification. (C) Amplification curve of the negative control. [file peerj-06-5993-s005.png]

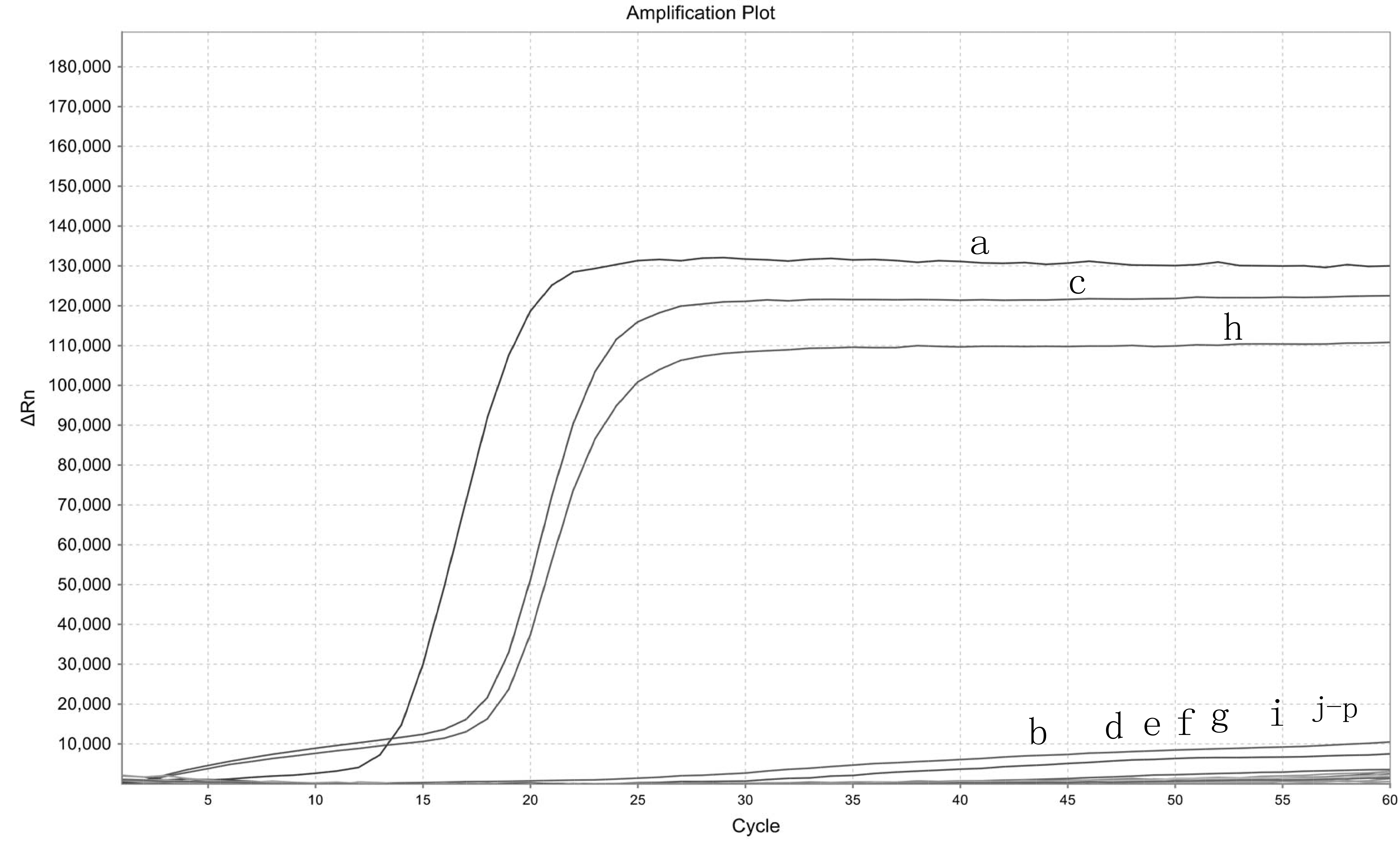

Supplement: Supplemental Information 6 — Clinical samples testing was carried out using the established real-time LAMP method. (A) Positive control (B) Negative control (C-P) Clinical samples 1–14. [file peerj-06-5993-s006.png]
